# Clinical sequencing uncovers the genomic characteristics and mutation spectrum of the 2018 African swine fever virus in Guangdong, China

**DOI:** 10.3389/fvets.2022.978243

**Published:** 2022-08-19

**Authors:** Zhi-ying Xu, Han Gao, Qi-yuan Kuang, Jia-bao Xing, Zhi-yuan Wang, Xin-yu Cao, Si-jia Xu, Jing Liu, Zhao Huang, Ze-zhong Zheng, Lang Gong, Heng Wang, Mang Shi, Gui-hong Zhang, Yan-kuo Sun

**Affiliations:** ^1^African Swine Fever Regional Laboratory of China, Guangzhou, China; ^2^Key Laboratory of Zoonosis Prevention and Control of Guangdong Province, College of Veterinary Medicine, South China Agricultural University, Guangzhou, China; ^3^Guangdong Laboratory for Lingnan Modern Agriculture, Guangzhou, China; ^4^National Engineering Research Center for Breeding Swine Industry, South China Agricultural University, Guangzhou, China; ^5^School of Medicine, Sun Yat-sen University, Guangzhou, China

**Keywords:** African swine fever, annotation, methylation, mutation spectrum, viral genome

## Abstract

African swine fever (ASF) outbreak have caused tremendous economic loss to the pig industry in China since its emergence in August 2018. Previous studies revealed that many published sequences are not suitable for detailed analyses due to the lack of data regarding quality parameters and methodology, and outdated annotations. Thus, high-quality genomes of highly pathogenic strains that can be used as references for early Chinese ASF outbreaks are still lacking, and little is known about the features of intra-host variants of ASF virus (ASFV). In this study, a full genome sequencing of clinical samples from the first ASF outbreak in Guangdong in 2018 was performed using MGI (MGI Tech Co., Ltd., Shenzhen, China) and Nanopore sequencing platforms, followed by Sanger sequencing to verify the variations. With 22 sequencing corrections, we obtained a high-quality genome of one of the earliest virulent isolates, GZ201801_2. After proofreading, we improved (add or modify) the annotations of this isolate using the whole genome alignment with Georgia 2007/1. Based on the complete genome sequence, we constructed the methylation profiles of early ASFV strains in China and predicted the potential 5mC and 6mA methylation sites, which are likely involved in metabolism, transcription, and replication. Additionally, the intra-host single nucleotide variant distribution and mutant allele frequency in the clinical samples of early strain were determined for the first time and found a strong preference for A and T substitution mutation, non-synonymous mutations, and mutations that resulted in amino acid substitutions into Lysine. In conclusion, this study provides a high-quality genome sequence, updated genome annotation, methylation profile, and mutation spectrum of early ASFV strains in China, thereby providing a reference basis for further studies on the evolution, transmission, and virulence of ASFV.

## Introduction

African swine fever (ASF) is a highly contagious viral disease in swine which exhibit a high mortality rate approaching 100% (1–3). ASF virus (ASFV), the causative agent of ASF, belongs to the order *Asfuvirales*, family *Asfarviridae*, and genus *Asfivirus* (4). ASFV was first introduced in northeast China in August 2018. Subsequently, the virus was isolated and analyzed (5, 6). Since then, ASFV has been causing series of outbreaks which resulted in significant economic loss nationwide (7). Recently, the emergence of ASFV strains with more genomic diversity and lower virulence in domestic pigs has been reported in China (8, 9).

To perform variant, transcriptome, and proteome analysis of ASFV, a high-quality genome is necessary (10). Several early strains, from the outbreaks in China, were isolated and sequenced using either Sanger sequencing or next-generation sequencing (NGS). However, these technologies could not overcome the limitations associated with sequencing large genomes, as the ASFV genome (170~193 kb) contains a series of homopolymers and repeat regions, including inverted terminal repeats of variable length (11, 12). Using data from three sequencing technologies, Nanopore, Illumina, and Sanger sequencing, 71 sequencing errors and several regional mistakes were corrected in the high-quality genome of the Georgia 2007/1 strain (13). Thus, there is still a need for a high-quality genome sequence for the ASFV strain that caused early outbreaks in China to be used as a reference for further research.

Intra-host single nucleotide variants (iSNVs), also known as quasispecies, are generated during virus evolutionary processes that occur within a host. Previous studies have reported that intra-host diversity of viral genomes was correlated with the virus virulence, immunogenicity, and infectivity (14). While consensus-level sequencing of ASFV is relatively commonplace in molecular epidemiology, few studies have explored the genetic characteristics of iSNVs and the mutational pool of ASFV. Currently, the characteristic of iSNVs of circulating ASFVs were found to be diverse unveiled by our lab (unpublished data). Especially, the characteristics of iSNVs of early strain are important and unavailable. It can serve as a reference for further studies on tracing the route, evolutionary patterns, and virulence of ASFV.

Herein, we used the MGI (MGI Tech Co., Ltd., Shenzhen, China) platform, Nanopore long-read sequencing, and Sanger sequencing to obtain a high-quality genome of the first virulent isolates of the ASFV in Guangdong. The genomic characteristics were also examined, including updated genomic annotations and predicted methylation modifications. Additionally, the iSNVs of this strain were explored. The results of this study provide in-depth knowledge of the early strains from the outbreak in China.

## Materials and methods

### Sample collection and virus isolation

Field samples were collected from the first ASF outbreak in Zhuhai, Guangdong Province, China in December 2018. Infected pigs showed 100% mortality. The spleens, lymph nodes, and other tissue specimens were collected from a single pig in one ASFV-positive herd. The virus was subsequently isolated from lymph nodes using porcine bone macrophages that were extracted from 4-week-old specific pathogen-free pigs (15). This virus was designated the name ASFV_GZ201801_2.

### DNA extraction and quantification

To avoid the accumulation of single nucleotide variants during the passage process, viral DNA for MGI platform sequencing was extracted directly from specific tissues. The first-generation culture of porcine bone macrophages was used to prepare extracellular virions for Nanopore sequencing. The QIAamp DNA Mini Kit (51304, Qiagen, Germany) was used for extraction, and the extracted viral DNA was purified using the AMPure XP Purification Kit (A63880, Beckman Coulter, USA). The quality and concentration were evaluated using Nanodrop Lite (ND-LITE-PR, ThermoFisher, USA) and Qubit 4.0 Fluorometer (Q33238, Invitrogen, USA). All these procedures were performed according to the manufacturer's instructions.

### Genomic sequencing

The purified viral DNA from clinical sample was processed using the MGI platform for genome sequencing to generate sequence data, whereas the DNA from the first-generation porcine bone macrophage culture viral supernatant was used for Nanopore sequencing, which was performed according to the manufacturer's instructions. Library preparation was carried out using the Ligation Sequencing Kit (SQK-LSK109, Oxford Nanopore Technologies, UK) and Native Barcoding Expansion 1–12 (EXP-NBD104, Oxford Nanopore Technologies, UK). Subsequently, the library was loaded onto the R9.4.1 MinION flow cell chip (FLO-MIN106, Oxford Nanopore Technologies, UK) and sequenced. Base calling was performed using Guppy v2.1.3 (Oxford Nanopore Technologies, UK).

### Analyses of sequencing reads

The data generated from the MGI platform were processed by removing adapter contamination and filtering using Trimmomatic v0.39 (16). Nanopore sequence data that had a quality value lower than Q20 were filtered using Fastp v0.20.1 (17). All filtered reads were hybrid-assembled using SPAdes v3.13.0 (18). The consensus sequence was subsequently generated from contigs, using iVar v1.3.1 (19). Subsequently, single nucleotide polymorphisms and indels were verified using bcftools v1.10.2 (20), and variants were validated by Sanger sequencing using clinical sample. Thereafter, genomic annotation was conducted using the Genome Annotation Transfer Utility (GATU) software; ASFV Georgia 2007/1 was used as the reference genome sequence (GenBank accession number: FR682468.2) (21).

### Genomic sequence analysis

To explore the genomic characteristics, ASFV references for genotype II, from GenBank, were downloaded and aligned using Mafft v7.471 (Table 1) (22). Multiple sequence alignment was performed and visualized using the R packages, RColorBrewer, and ggplot2 (23, 24). Nanopore fast5 files were used to screen potentially methylated nucleotides using Tombo v1.5.1 (25). Andefault model of Tombo was automatically selected to detect 5mC and 6mA methylation modifications in the ASFV_GZ201801_2 genome, and it subsequently computed the corresponding scores.

**Table 1:** Reference strains selected in this study.

**Name**	**GenBank ID**	**Country/province**	**Isolate year**	**Length (bp)**
Georgia 2007/1	FR682468.2	Georgia	2007	190,584
China/2018/AnhuiXCGQ	MK128995.1	Anhui	2018	189,393
pig/HLJ/2018	MK333180.1	Heilongjiang	2018	189,404
DB/LN/2018	MK333181.1	Liaoning	2018	189,404
pig/China/CAS19-1/2019	MN172368.1	Guangdong	2019	189,405
Wuhan 2019-1	MN393476.1	Hubei	2019	190,576
Wuhan 2019-2	MN393477.1	Hubei	2019	190,576
CADC_HN09	MZ614662.1	China	2019	190,257
pig/Heilongjiang/HRB1/2020	MW656282.1	Heilongjiang	2020	189,355
WBBS01	MK645909.1	China	2018	189,394
CN/2019/InnerMongolia-AES01	MK940252.1	Inner Mongolia	2019	189,403
SY18	MH766894.2	Liaoning	2018	188,643
HuB20	MW521382.1	Hubei	2020	188,643
GZ201801	MT496893.1	Guangdong	2018	189,393

### Calling of iSNVs

The genome sequence that was validated by Sanger sequencing was used for iSNV analysis. Clean reads with high-quality bases (Q>20) were mapped to the validated genome sequence using BWA v0.7.17 (26) and then reformatted using SAMtools v1.10 (27). Subsequently, iVar v1.3.1, mentioned above, was then used to call iSNVs. Several criteria were used to screen for high-quality iSNVs: (1) total sequencing depth at iSNV site ≥100, (2) minor allele frequency ≥1%, and (3) depth of the minor allele ≥5. iSNVs that met the criteria were counted. To analyze the characteristics of the iSNVs, we calculated the preference of mutation types, the position of mutation, and the effect of mutation in the corresponding amino acid or ORF (Open Reading Frame).

## Results

### Improved sequence obtained by hybrid assembly of sequencing data

**Figure 1:**
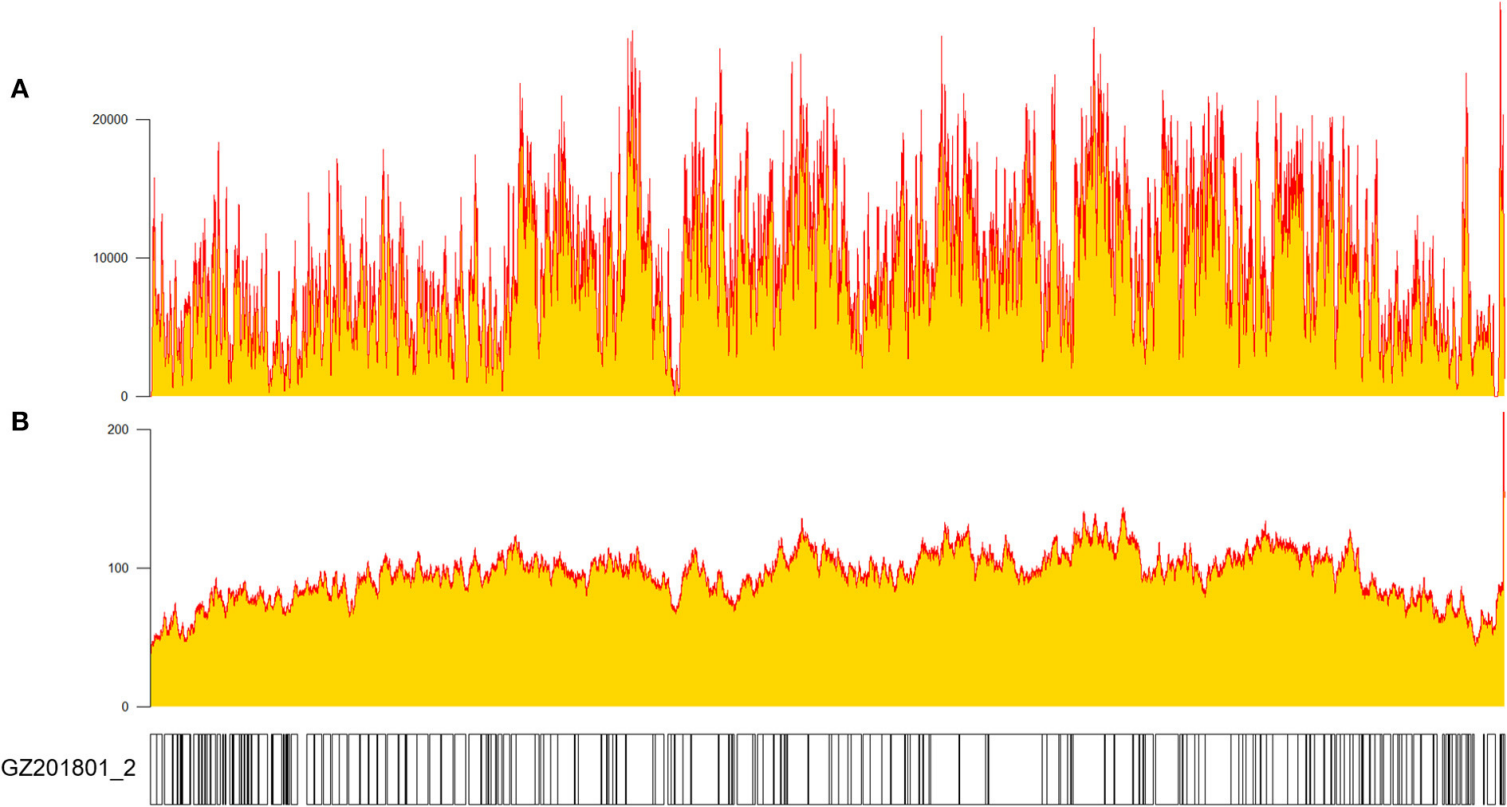
Depth and coverage of GZ201801_2 measured using SAMtools v1.10 after sequencing (27). **(A)** MGI platform sequencing and **(B)** Nanopore sequencing.

The MGI/Nanopore sequencing data was processed, and the variants were identified using Sanger sequencing. Combining the advantage of MGI (High throughput), Nanopore (Long-read) and Sanger sequencing (High accuracy), we obtained a high-quality genome, named ASFV_GZ201801_2 (Genbank ID: ON263123). The genome sequence of this strain had 189,401 base pairs (bp) (Figure 1), with a high depth (MGI mean depth: 6656×, Nanopore mean depth: 74×) and total coverage (100%). Data from Nanopore platform were distributed evenly in the whole genome, and hybrid of sequencing data from two platforms led to a good resolution of ASFV genome. Compared our genome with early ASFV representative strains in China (ASFV Pig/HLJ/18: MK333180.1, ASFV China/2018/AnhuiXCGQ: MK128995.1), similar genome sizes found. ASFV_GZ201801_2 was annotated using ASFV Georgia 2007/1 as the reference genome. Furthermore, the novel annotations of this strain were explored.

### Updated annotations on the basic of ASFV Georgia 2007/1

**Figure 2:**
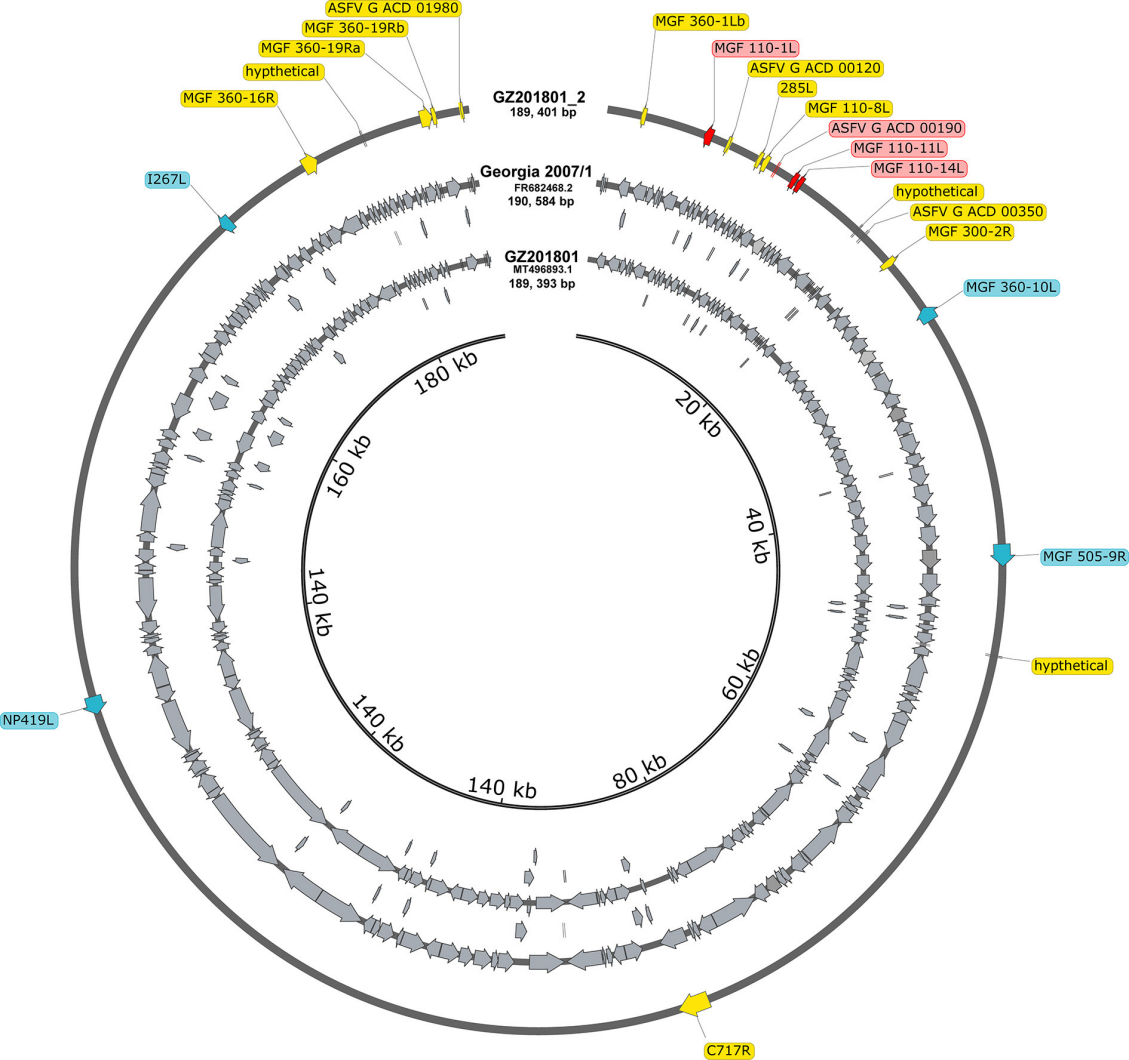
All annotations in the entire genome of Georgia 2007/1 and GZ201801. Compared to GZ201801, the genes marked with yellow represent the updated annotation of the GZ201801_2. The genes in blue and red represent different annotations GZ201801_2 in Georgia 2007/1, where blue represents different annotations due to site mutations, and red represents different annotations that appear owing to deletion or early termination.

The annotations were manually examined and modified to fit any changes in ORFs, such as splits, fusions, or truncations. A total of 194 annotations were identified, eight of which were modified to accommodate changes caused by indels and nucleotide substitutions in the ORFs of ASFV_GZ201801_2. Modified annotations included the following ORFs: *MGF 110-1L, ASFV G ACD 00190, MGF 110-11L, MGF 110-14L, MGF 360-10L, MGF 505-9R, NP419L*, and *I267L*. The verified ASFV_GZ201801_2, comparing with ASFV_GZ201801 (179 ORFs), had clarified 14 novel annotations, which were *285L, ASFV_G_ACD_00120, ASFV_G_ACD_00350, ASFV_G_ACD_01980, C717R, hypothetical, hypothetical1, hypothetical2, MGF_100-3L, MGF_300-2R, MGF_360-16R, MGF_360-1Lb, MGF_360-19Ra*, and *MGF_360-19Rb* (Figure 2). Notably, a series of genes in ASFV Georgia 2007/1 were annotated without the ATG initiation codon: *DP60R, ASFV_G_ACD_00190, ASFV_G_ACD_00270, ASFV_G_ACD_00350, MGF_360-15R, NP1450L*, and *ASFV_G_ACD_01980*, which started with leucine (UUA/UUG), while *MGF_110-8L* initiated with valine (GTG).

### Genomic characteristic of ASFV_GZ201801_2

**Figure 3:**
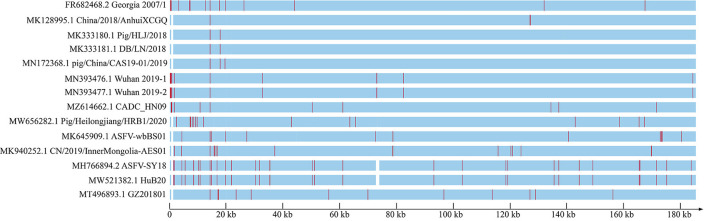
ASFV genome from China and Georgia 2007/1, which has been considered as the original strain circulating around Eurasia since 2007, showing the entire genome frame map of all genome-wide sequences compared to GZ201801_2. Each red line represents a site mutation, and the vacant positions represent deletion regions.

To explore the genomic characteristics of ASFV_GZ201801_2, we aligned all the available ASFV whole genome sequences in China and ASFV Georgia 2007/1 that circulated in Eastern Europe, for more than a decade ago, with the improved ASFV_GZ201801_2. ASFV GZ201801_2, ASFV Georgia 2007/1, and other early strains shared several indels and substitutions, which led to high overall nucleotide identification (from 98.3% to approximately 100%). The 5′-end of the genome of ASFV_GZ201801_2 exhibited characteristics similar to those of ASFV Georgia 2007/1 (FR682468.2); however, several isolates from China demonstrated homogeneous 5′-end deletions, except for three strains (MN393476.1, MN393477.1, and MZ614662.1). We further compared our verified sequence to ASFV Pig/HLJ/2018 and found 5 indels and 6 substitutions located in homopolymer regions, which may be associated with sequencing errors. Similar findings were also observed in several other early strains from China. Furthermore, we demonstrated that the recent strains, in China, had undergone more variations and showed more diversity than earlier strains (Figure 3).

### Potential 5mC and 6mA methylation

**Figure 4:**
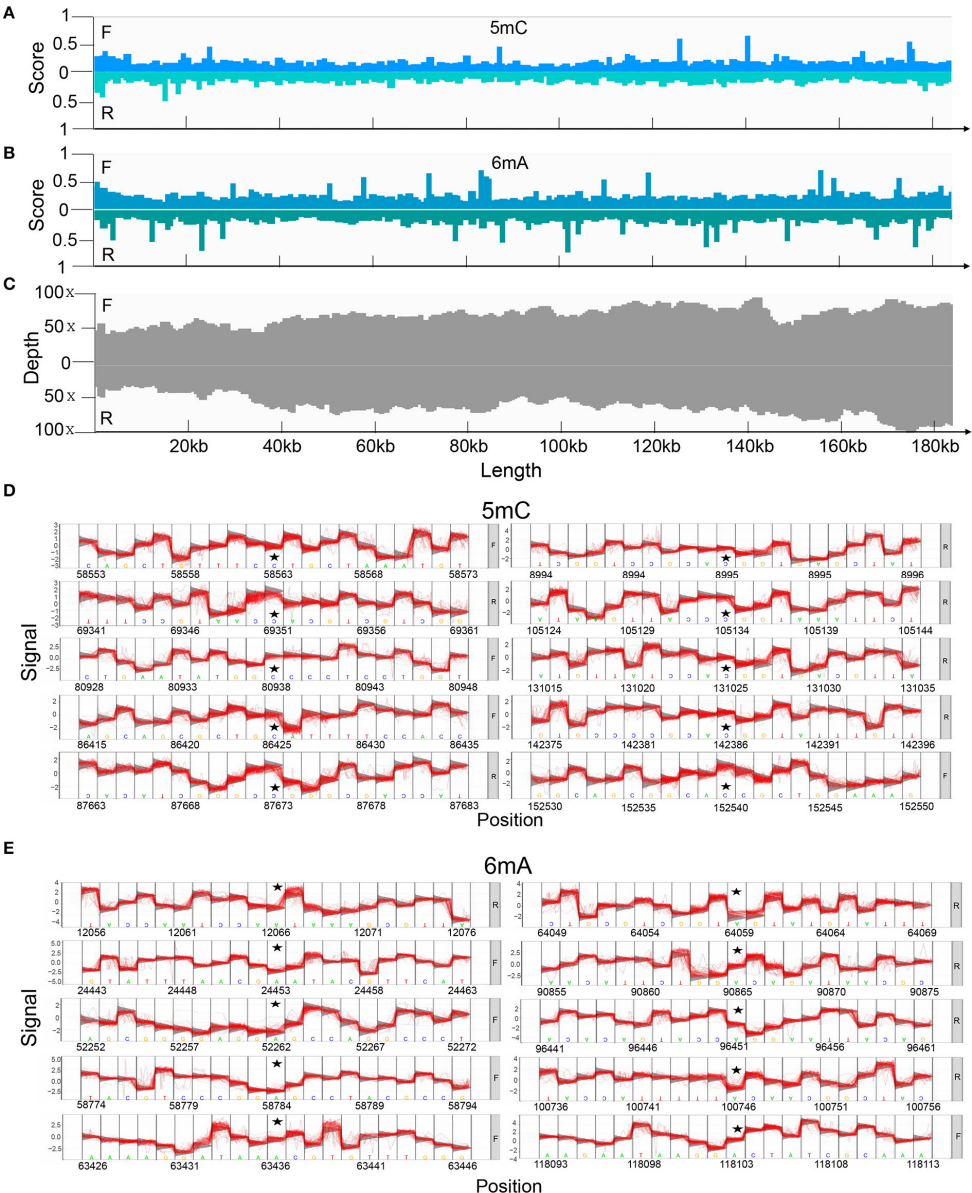
Methylation of positive and reverse strands in the genome predicted using Tombo and its corresponding depth. **(A)** The score of 5mC and **(B)** 6mA modification of positive and reverse strands. We compared the raw signal levels between sequenced samples and alternative models to obtain the statistical results of the corresponding sites, Tombo generated the correlation scores to evaluate the possibility of methylation on certain sites in the full-length viral genome. A higher score indicated that the site was more likely to undergo methylation modification. **(C)** Depth of Nanopore sequencing of positive and reverse strands. The top 10 methylation sites and their adjacent regions in the ASFV_GZ201801_2 that are most likely to be modified by **(D)** 5mC and **(E)** 6mA methylation also predicted using Tombo.

Potential 5mC and 6mA methylation were analyzed using Nanopore data. The methylation sites were predicted to be evenly distributed along the genome without remarkable regional preferences. In addition, a similar methylation state on the forward and reverse strands was noted, and the 6mA methylation scores at various sites on the entire genome were generally greater than those of 5mC methylation (Figures 4A–C). We predicted ten methylation sites, including their adjacent regions, in ASFV_GZ201801_2 that were most likely to have 5mC and 6mA modifications (Figures 4D,E). The genes that showed significant 5mC methylation included *F778R, EP1242L, M448R, C257L, C475L, C962R, B646L, NP1450L, D1133L*, and *H359L*, while the genes that showed significant 6mA methylation were *MGF_100-1R, MGF_360-8L, A859L, F778R, F1055L, K205R, C962R, B318L, B354L*, and *CP2475L*. The genes, including the potential 5mC and 6mA methylation sites, were primarily involved in metabolism, transcription, and replication (Tables 2, 3) (28).

**Table 2:** Gene position, coded protein, and biological processes of the top 10 sites where 5mC methylation modification was predicted (28).

**ORF**	**Position**		**Function**	**Biological processes**
	**Start**	**Stop**		
F778R	57,745	60,081	Ribonucleotide reductase (large subunit)	Nucleotide metabolism, transcription, replication, and repair
EP1242L	67,263	70,991	RNA polymerase subunit 2	Nucleotide metabolism, transcription, replication, and repair
M448R	80,232	81,578	Uncharacterized protein	Unknown
C257L	85,706	86,479	Uncharacterized protein	Transmembrane domain
C475L	86,458	87,885	Poly-A polymerase large subunit	Nucleotide metabolism, transcription, replication, and repair
C962R	89,826	92,714	DNA primase	Nucleotide metabolism, transcription, replication, and repair
B646L	104,342	106,282	P72 major capsid protein	Structural integrity: structural proteins and proteins involved in morphogenesis
NP1450L	129,825	134,207	RNA polymerase subunit 1	Nucleotide metabolism, transcription, replication, and repair
D1133L	141,100	144,501	Helicase superfamily II	Nucleotide metabolism, transcription, replication, and repair
H359L	151,901	152,980	RNA polymerase subunit 3–11	Nucleotide metabolism, transcription, replication, and repair

**Table 3:** Gene position, encoded protein, and biological process of the top 10 sites where the 6mA methylation was predicted (28–30).

**ORF**	**Position**		**Function**	**Biological processes**
	**Start**	**Stop**		
MGF 100-1R	11,823	12,197	Uncharacterized protein	Nucleotide metabolism, transcription, replication, and repair
MGF 360-8L	23,778	24,737	Uncharacterized protein	Unknown
A859L	51,945	54,521	RNA helicase	Nucleotide metabolism, transcription, replication, and repair
F778R	57,745	60,081	Ribonucleotide reductase (large subunit)	Nucleotide metabolism, transcription, replication, and repair
F1055L	60,598	63,744	Uncharacterized protein	Nucleotide metabolism, transcription, replication, and repair
K205R	63,938	64,555	Related to ER stress	Activates ER stress, autophagy, and NF-κb signaling pathway
C962R	89,826	92,714	DNA primase	Nucleotide metabolism, transcription, replication, and repair
B318L	96,040	96,996	Prenyltransferase	Enzymes
B354L	100,365	101,429	Uncharacterized protein	Unknown
CP2475L	118,014	125,444	pp.220 polyprotein precursor of p150, p37, p14, and p34. required for packaging of nucleoprotein core	Structural integrity: structural proteins and proteins involved in morphogenesis

### Identification of intra-host variations in ASFV_GZ201801_2

**Figure 5:**
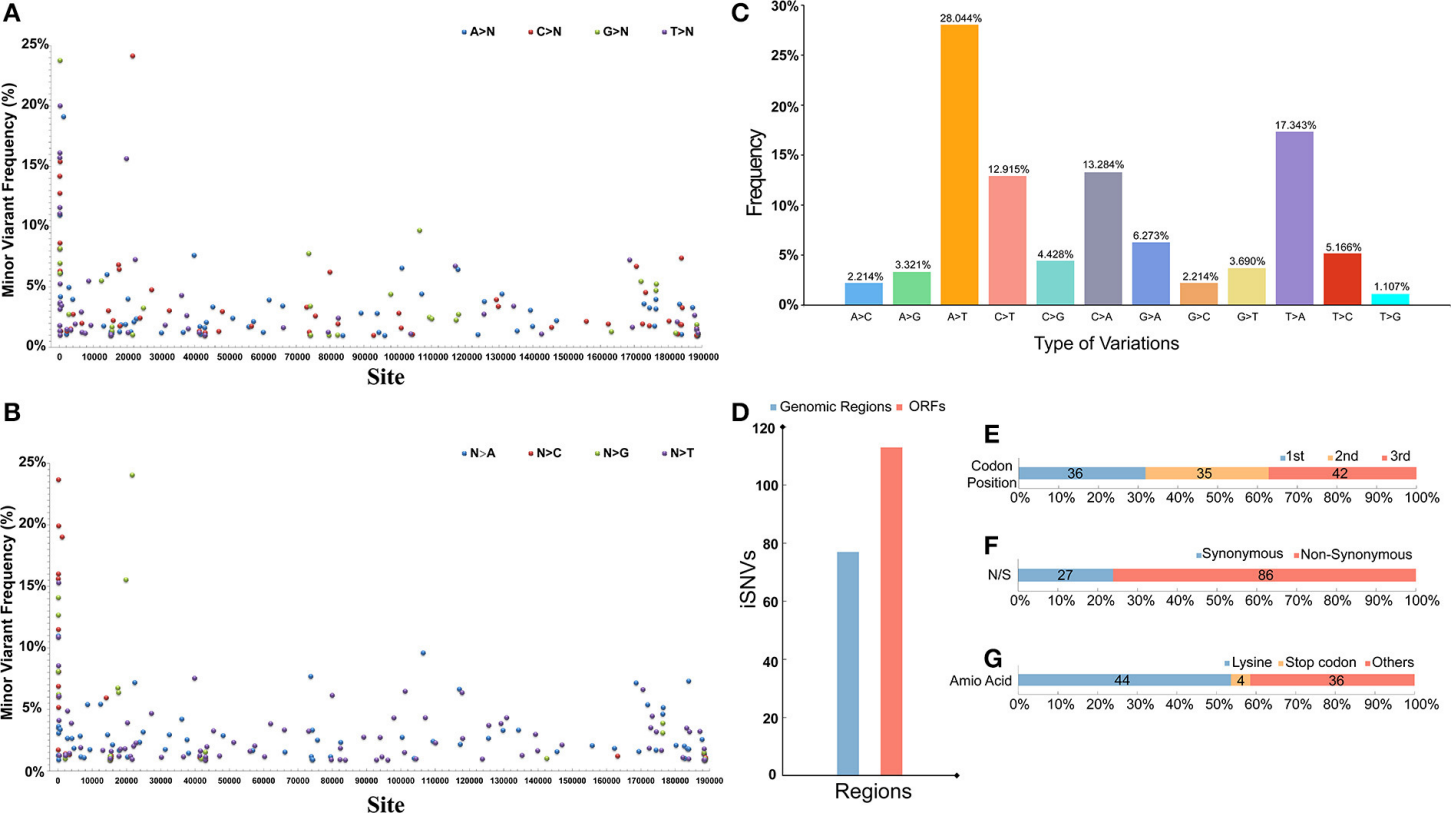
**(A)** The distribution and frequency of theiSNVs) that a certain base (A, T, C, G) mutate into other three bases. **(B)** The distribution and frequency of the iSNVs that the other three bases mutate into a certain base. **(C)** The percentage of different iSNVs mutation types. **(D)** Number of iSNVs in coding and non-coding regions. **(E)** Proportion of iSNVs occurring in different positions in codons across the genome. **(F)** Proportion of iSNVs causing non-synonymous and synonymous mutations. **(G)** Proportion of iSNVs that mutate into different type of amino acid. The number of iSNVs in each figure is indicated on the corresponding bar.

Through the deep MGI sequencing of the genomic DNA, extracted from the clinical specimens, we achieved a high sequencing depth for all sites across the genome, with a mean depth of 6656 (coverage > 99%). Minor iSNVs were called by setting the threshold for minor allele frequency at 1% to exclude potential sequencing errors and minimize false discovery rates. The viral populations revealed a low frequency (<25%) cloud of iSNVs detectable across the entire genome. The minor iSNVs were distributed evenly on the genome, and marginally higher mutation frequencies were observed at the 5'-end than in the rest of the genome. Moreover, mutation preference across the genome was explored. To be specific, no obvious mutation preference could be observed when we calculated the mutations from a certain base to the other three bases (Figure 5A). On the contrary, when we calculated the mutations from the other three bases to a certain base, strong preference was found in Adenine (A) and Thymine (T) (Figure 5B). We further calculated the types of mutations in all the bases and their frequency and found that substitutions involving A and T occupied the largest proportion, reaching 28.044 and 17.343%, respectively. In addition, other mutations substituting Cytosine (C) with T or A occupied 10% above, whereas the rest of the mutations showed low mutation frequencies (Figure 5C). We further investigated the regions where the mutations were located, and 113 of the 190 iSNVs were found in the coding region (Figure 5D). Among all the genes, iSNVs were found slightly enrich in *MGF 360-13La, MGF 505-7R, I96L*, and *MGF 360-21R*. Moreover, the mutations were evenly distributed in the three positions of the codons which encode an amio acid, and the proportion of non-synonymous mutations was 76.11%, which was three times that of synonymous mutations (Figures 5E,F). More than half of the non-synonymous mutations showed a preference for lysine. Notably, 4 iSNVs of the non-synonymous mutations preferred to occur in the stop codon, which caused a frameshift mutation in the entire ORF; the four iSNVs were located in *ASFV G ACD 00120, MGF 505-4R, K421R*, and *EP153R* (Figure 5G).

## Discussion

ASFV has been circulating in China for 5 years. Recently, low virulent strains with mutations and indels in the genomes have been detected; these strains have varying biological characteristics (8). The identification of mutations and deletions requires a high-quality genome. However, in China, there is still a lack of high-quality genomes of the early strains to use as references when studying the long-term effects of deletions and mutations. The complete genome of ASFV is typically obtained using either Sanger sequencing or NGS (11, 31). However, genome sequences generated from either one of these technologies do not reflect the actual genomic characteristics of ASFV, which has a large and intricate genome (13). The co-employment of Nanopore sequencing and NGS is an emerging strategy that is used to acquire complex microbial genomes, which combine the advantage of long sequencing reads from Nanopore and high accuracy from NGS (32, 33). Here, we combined the data generated from the MGI sequencing platform, Nanopore platforms, and Sanger sequencing to verify and obtain a high-quality genome of the lethal ASFV isolate, ASFV_GZ201801_2, which we collected during the early phase of the ASF pandemic in China.

Because of its complex genome, many genomic characteristics of ASFV remain unknown; thus, an accurate genome sequence was urgently needed. We compared ASFV_GZ201801_2 and ASFV Georgia 2007/1(FR682468.2) and detected a large number of site mutations, which had accumulated over the past decade. Furthermore, the genome sequence that we acquired, using samples from the first case of a high virulence ASF outbreak in China, could reflect the real epidemic strains from the early ASFV outbreak. In addition, we compared ASFV_GZ201801_2, ASFV Pig/HLJ/2018, and ASFV China/2018/AnhuiXCGQ and found that the strains from the early outbreaks in China shared a few same mutations. To conduct molecular epidemiology investigations of ASFV in China, a high-quality genome, isolated from the early strains from the first outbreak, is needed for reference. Many new site mutations have appeared in the recent Chinese strains, indicating the growing diversity of the ASFV strains in China. However, current circulating strains have shared several doubtful mutations when compared with early reference genome sequences, which may be caused by sequencing errors. Lacking a preferred reference would hinder further epidemiological studies of ASFV in China. Our verified genome sequence could be utilized as a reference for the early strains to trace the transmission route and evolution of ASFV.

Globally, the annotation of ASFV is constantly being updated; however, annotations of Chinese ASFV isolates still need to be confirmed. In our study, we found outdated and ambiguous annotations. We refined our annotations using the well-known sequence, ASFV Georgia 2007/1; several annotations were modified to accommodate changes in our genome. However, owing to the lack of transcriptome and proteome studies, the expression data of these ORFs was insufficient. There is a need to validate ASFV genome annotations reported by different studies worldwide.

Methylation of specific viral genes is involved in the transition from lytic to latent infection. Viral DNA methylation plays a crucial role in the life cycle of Epstein-Barr virus, adenovirus, and hepatitis B virus (34–38). Whether ASFV genome has DNA methylation and epigenetic regulation is to be discerned. There was research claimed that there is no ASFV methylation in infected Vero cells, while previous studies of the ASFV BA71V strain revealed the methylation of its 5′ cap (39, 40). With the development of technology, the methylation ASFV Wuhan 2019/1 was predicted using Nanopore which could detect methylation in high accuracy (41). Based on several recent clinical reports of ASF outbreaks in China, the period of onset of ASF in ASFV-positive pigs had extended, which might be a consequence of distinct ASFV DNA methylation. The mechanism related to epigenetic modifications underlying this phenomenon remains unclear and requires further exploration. Furthermore, previous studies have reported that 5mC and 6mA modifications potentially exist in the ASFV genome; these may be essential for virus-host interactions (41). In summary, our research contributes to the elucidation of the methylation profile of early AFSV strains in China using Nanopore sequencing technology, which provides a foundation for further research on the ASFV life cycle and immune regulation.

The iSNVs detected in early strains can be treated as the advantage mutation pool of ASFV, from which gain (or lose)-of-function mutations may be acquired by current or future epidemic strains; these iSNVs provide valuable information for epidemiology investigation and viral evolution studies. Eighty-five percent of the amino acid variations of the current SARS-CoV-2 genome correspond with iSNVs found in the early strains, which suggested that in-depth studies of the mutation pool of early strains were necessary. To characterize the landscape of within-host variants of early ASFV strains, we employed the deep MGI sequencing of viral DNA, extracted from ASFV-positive samples, to reveal the mutation spectrum, which may be used as a reference for current and future studies. The frequency of iSNVs in the ASFV genome was low, below 25%, which indicated a slow mutation rate. The frequency of substitution mutations, substituting with A or T, was substantially higher than the others. This indicated that the replication of ASFV showed preference, and the mechanism and effects of the replication process need to be further studied. The mutations were more concentrated in the coding region than in the non-coding region. Considering that the length of the coding region in ASFV is approximately 8.5 times the length of the non-coding region, the probability of mutation in the non-coding region is considerably higher than that of the coding region, which may be related to the regulation of ASFV. The regulatory role of the non-coding region between genes was previously reported (42), which also suggests that future research on ASFV should focus on the non-coding region. In addition, mutations have a similar probability of occurring at each location in the codons with no significant bias, which indicates that the choice of mutation does not favor a certain base in the codon. The probability of non-synonymous mutations is considerably higher than that of synonymous mutations. Non-synonymous mutations typically disappear owing to the effects of population selection, but their accumulation can lead to dramatic changes in the virulence, immunogenicity, and infectivity of the virus (43, 44), and afford an opportunity for viral adaptation (45). More than half of these non-synonymous mutations resulted in substitution with lysine, which is encoded by AAA. This mutation is commonly found near consecutive bases, which are widely distributed in the ASFV genome, but the mechanism underlying the preference for this type of mutation requires further investigation. To our knowledge, this is the first time that the mutation spectrum of early ASFV strains was determined. Our findings provide a reference to use in further investigations on molecular epidemiology, evolution, host adaptation, and pathogenicity of ASFV.

## Conclusions

To our knowledge, this is the first time that the high-quality proofread genome sequence of GZ201801_2 and the mutant spectrum of early strains of ASFV were revealed. Our findings would provide a reference to pave the way for further investigations on molecular epidemiologic investigations, evolution, host adaptation, and pathogenicity of ASFV.

## Data availability statement

The datasets presented in this study can be found in online repositories. The names of the repository/repositories and accession number(s) can be found at: https://www.ncbi.nlm.nih.gov/genbank/, ON263123.

## Author contributions

Z-yX: writing original draft and software. HG: writing review and editing. Q-yK: data curation and validation. J-bX: visualization and validation. Z-yW: data curation. X-yC: validation. S-jX: formal analysis. JL: investigation. ZH and MS: methodology. Z-zZ: resources. LG: funding acquisition. HW: supervision. G-hZ and Y-kS: resources and project administration. All authors contributed to the article and approved the submitted version.

## Conflict of interest

The authors declare that the research was conducted in the absence of any commercial or financial relationships that could be construed as a potential conflict of interest.

## Publisher's note

All claims expressed in this article are solely those of the authors and do not necessarily represent those of their affiliated organizations, or those of the publisher, the editors and the reviewers. Any product that may be evaluated in this article, or claim that may be made by its manufacturer, is not guaranteed or endorsed by the publisher.

## References

[1] Wang G Xie M Wu W Chen Z. Structures and functional diversities of ASFV proteins. Viruses. (2021) 13:2124. 10.3390/v13112124 34834930 PMC8619059

[2] Gaudreault NN Madden DW Wilson WC Trujillo JD Richt JA. African swine fever virus: an emerging DNA arbovirus. Front Vet Sci. (2020) 7:215. 10.3389/fvets.2020.00215 32478103 PMC7237725

[3] Olesen AS Lohse L Boklund A Halasa T Gallardo C Pejsak Z. Transmission of African swine fever virus from infected pigs by direct contact and aerosol routes. Vet Microbiol. (2017) 211:92–102. 10.1016/j.vetmic.2017.10.004 29102127

[4] Guo F Shi Y Yang M Guo Y Shen Z Li M. The structural basis of African swine fever virus core shell protein p15 binding to DNA. FASEB J. (2021) 35:e21350. 10.1096/fj.202002145R 33629764

[5] Wen X He X Zhang X Zhang X Liu L Guan Y. Genome sequences derived from pig and dried blood pig feed samples provide important insights into the transmission of African swine fever virus in China in 2018. Emerg Microbes Infect. (2019) 8:303–6. 10.1080/22221751.2019.1565915 30866784 PMC6455166

[6] Zhou X Li N Luo Y Liu Y Miao F Chen T. Emergence of African swine fever in China, 2018. Transbound Emerg Dis. (2018) 65:1482–4. 10.1111/tbed.12989 30102848

[7] Zhao D Liu R Zhang X Li F Wang J Zhang J. Replication and virulence in pigs of the first African swine fever virus isolated in China. Emerg Microbes Infect. (2019) 8:438–47. 10.1080/22221751.2019.1590128 30898043 PMC6455124

[8] Sun E Huang L Zhang X Zhang J Shen D Zhang Z. Genotype I African swine fever viruses emerged in domestic pigs in China and caused chronic infection. Emerg Microbes Infect. (2021) 10:2183–93. 10.1080/22221751.2021.1999779 34709128 PMC8635679

[9] Sun E Zhang Z Wang Z He X Zhang X Wang L. Emergence and prevalence of naturally occurring lower virulent African swine fever viruses in domestic pigs in China in 2020. Sci China Life Sci. (2021) 64:752–65. 10.1007/s11427-021-1904-4 33655434

[10] Forth JH Forth LF Blome S Hoper D Beer M. African swine fever whole-genome sequencing-quantity wanted but quality needed. PLoS Pathog. (2020) 16:e1008779. 10.1371/journal.ppat.1008779 32853289 PMC7451517

[11] Bao J Wang Q Lin P Liu C Li L Wu X. Genome comparison of African swine fever virus China/2018/AnhuiXCGQ strain and related European p72 genotype II strains. Transbound Emerg Dis. (2019) 66:1167–76. 10.1111/tbed.13124 30637968

[12] Dixon LK Sun H Roberts H. African swine fever. Antiviral Res. (2019) 165:34–41. 10.1016/j.antiviral.2019.02.018 30836106

[13] Forth JH Forth LF King J Groza O Hubner A Olesen AS. A deep-sequencing workflow for the fast and efficient generation of high-quality African swine fever virus whole-genome sequences. Viruses. (2019) 11:846. 10.3390/v11090846 31514438 PMC6783980

[14] Andersen KG Shapiro BJ Matranga CB Sealfon R Lin AE Moses LM. Clinical sequencing uncovers origins and evolution of lassa virus. Cell. (2015) 162:738–50. 10.1016/j.cell.2015.07.020 26276630 PMC4537774

[15] Carrillo C Borca MV Afonso CL Onisk DV Rock DL. Long-term persistent infection of swine monocytes/macrophages with African swine fever virus. J Virol. (1994) 68:580–3. 10.1128/jvi.68.1.580-583.1994 8254776 PMC236326

[16] Bolger AM Lohse M Usadel B. Trimmomatic: a flexible trimmer for Illumina sequence data. Bioinformatics. (2014) 30:2114–20. 10.1093/bioinformatics/btu170 24695404 PMC4103590

[17] Chen S Zhou Y Chen Y Gu J. fastp: an ultra-fast all-in-one FASTQ preprocessor. Bioinformatics. (2018) 34:i884–90. 10.1093/bioinformatics/bty560 30423086 PMC6129281

[18] Bankevich A Nurk S Antipov D Gurevich AA Dvorkin M Kulikov AS. SPAdes: a new genome assembly algorithm and its applications to single-cell sequencing. J Comput Biol. (2012) 19:455–77. 10.1089/cmb.2012.0021 22506599 PMC3342519

[19] Castellano S Cestari F Faglioni G Tenedini E Marino M Artuso L. iVar, an interpretation-oriented tool to manage the update and revision of variant annotation and classification. Genes. (2021) 12:384. 10.3390/genes12030384 33800487 PMC8001268

[20] Danecek P Bonfield JK Liddle J Marshall J Ohan V Pollard MO. Twelve years of SAMtools and BCFtools. Gigascience. (2021) 10:giab008. 10.1093/gigascience/giab008 33590861 PMC7931819

[21] Tcherepanov V Ehlers A Upton C. Genome Annotation Transfer Utility (GATU): rapid annotation of viral genomes using a closely related reference genome. BMC Genomics. (2006) 7:150. 10.1186/1471-2164-7-150 16772042 PMC1534038

[22] Katoh K Rozewicki J Yamada KD. MAFFT online service: multiple sequence alignment, interactive sequence choice and visualization. Brief Bioinform. (2019) 20:1160–6. 10.1093/bib/bbx108 28968734 PMC6781576

[23] Neuwirth E. RColorBrewer: ColorBrewer Palettes. (2014). Available online at: https://rdrr.io/cran/RColorBrewer/man/ColorBrewer.html

[24] Wickham H. ggplot2: Elegant Graphics for Data Analysis. New York, NY: Springer-Verlag (2016).

[25] Stoiber M Quick J Egan R Eun Lee J Celniker S Neely RK. De novo identification of DNA modifications enabled by genome-guided nanopore signal processing. bioRxiv. (2017). 10.1101/094672

[26] Jo H Koh G. Faster single-end alignment generation utilizing multi-thread for BWA. Biomed Mater Eng. (2015) 26:S1791–6. 10.3233/BME-151480 26405948

[27] Li H Handsaker B Wysoker A Fennell T Ruan J Homer N. The sequence alignment/map format and SAMtools. Bioinformatics. (2009) 25:2078–9. 10.1093/bioinformatics/btp352 19505943 PMC2723002

[28] Dixon LK Chapman DA Netherton CL Upton C. African swine fever virus replication and genomics. Virus Res. (2013) 173:3–14. 10.1016/j.virusres.2012.10.020 23142553

[29] Wang Q Zhou L Wang J Su D Li D Du Y. African swine fever virus K205R induces ER stress and consequently activates autophagy and the NF-kappaB signaling pathway. Viruses. (2022) 14:394. 10.3390/v14020394 35215987 PMC8880579

[30] Ramirez-Medina E Vuono EA Pruitt S Rai A Espinoza N Velazquez-Salinas L. Evaluation of an ASFV RNA helicase gene A859L for virus replication and swine virulence. Viruses. (2021) 14:10. 10.3390/v14010010 35062213 PMC8777736

[31] Wang X Wang X Zhang S He Y Chen X Liu X. Genetic characterization and variation of African swine fever virus China/GD/2019 strain in domestic pigs. Pathogens. (2022) 11:97. 10.3390/pathogens11010097 35056045 PMC8780551

[32] McNaughton AL Roberts HE Bonsall D de Cesare M Mokaya J Lumley SF. Illumina and Nanopore methods for whole genome sequencing of hepatitis B virus (HBV). Sci Rep. (2019) 9:7081. 10.1038/s41598-019-43524-9 31068626 PMC6506499

[33] Kafetzopoulou LE Efthymiadis K Lewandowski K Crook A Carter D Osborne J. Assessment of metagenomic Nanopore and Illumina sequencing for recovering whole genome sequences of chikungunya and dengue viruses directly from clinical samples. Euro Surveill. (2018) 23:1800228. 10.2807/1560-7917.ES.2018.23.50.1800228 30563591 PMC6299504

[34] Watanabe Y Yamamoto H Oikawa R Toyota M Yamamoto M Kokudo N. DNA methylation at hepatitis B viral integrants is associated with methylation at flanking human genomic sequences. Genome Res. (2015) 25:328–37. 10.1101/gr.175240.114 25653310 PMC4352876

[35] Koumbi L Karayiannis P. The epigenetic control of Hepatitis B virus modulates the outcome of infection. Front Microbiol. (2015) 6:1491. 10.3389/fmicb.2015.01491 26779147 PMC4701982

[36] Hoelzer K Shackelton LA Parrish CR. Presence and role of cytosine methylation in DNA viruses of animals. Nucleic Acids Res. (2008) 36:2825–37. 10.1093/nar/gkn121 18367473 PMC2396429

[37] Tao Q Robertson KD. Stealth technology: how Epstein-Barr virus utilizes DNA methylation to cloak itself from immune detection. Clin Immunol. (2003) 109:53–63. 10.1016/S1521-6616(03)00198-0 14585276

[38] Ambinder RF Robertson KD Tao Q. DNA methylation and the Epstein-Barr virus. Semin Cancer Biol. (1999) 9:369–75. 10.1006/scbi.1999.0137 10547345

[39] Salas ML Kuznar J Vinuela E. Polyadenylation, methylation, and capping of the RNA synthesized *in vitro* by African swine fever virus. Virology. (1981) 113:484–91. 10.1016/0042-6822(81)90176-8 6168100

[40] Weber S Hakobyan A Zakaryan H Doerfler W. Intracellular African swine fever virus DNA remains unmethylated in infected Vero cells. Epigenomics. (2018) 10:289–99. 10.2217/epi-2017-0131 29327614

[41] Jia L Chen J Liu H Fan W Wang D Li J. Potential m6A and m5C methylations within the genome of a Chinese African swine fever virus strain. Virol Sin. (2021) 36:321–4. 10.1007/s12250-020-00217-2 32270427 PMC8087734

[42] Dunn LEM Ivens A Netherton CL Chapman DAG Beard PM. Identification of a functional small noncoding RNA of African swine fever virus. J Virol. (2020) 94:e01515–20. 10.1128/JVI.01515-20 32796064 PMC7565616

[43] Ladner JT Wiley MR Mate S Dudas G Prieto K Lovett S. Evolution and spread of Ebola virus in Liberia, 2014–2015. Cell Host Microbe. (2015) 18:659–69. 10.1016/j.chom.2015.11.008 26651942 PMC4711363

[44] Illingworth CJ Fischer A Mustonen V. Identifying selection in the within-host evolution of influenza using viral sequence data. PLoS Comput Biol. (2014) 10:e1003755. 10.1371/journal.pcbi.1003755 25080215 PMC4117419

[45] Gire SK Goba A Andersen KG Sealfon RS Park DJ Kanneh L. Genomic surveillance elucidates Ebola virus origin and transmission during the 2014 outbreak. Science. (2014) 345:1369–72. 10.1126/science.1259657 25214632 PMC4431643

